# *In vitro* biological screening of a critically endangered medicinal plant, *Atropa acuminata Royle Ex Lindl* of north western Himalaya

**DOI:** 10.1038/s41598-018-29231-x

**Published:** 2018-07-23

**Authors:** Khaista Rahman, Shahid Ullah Khan, Shah Fahad, Zabta Khan Shinwari, Dilfaraz Khan, Sajid Kamal, Ikram Ullah, Syed Ishtiaq Anjum, Shad Man, Abdul Jamil Khan, Wasim Ullah Khan, Muhammad Hafeez Ullah Khan, Mehmood Jan, Muhammad Adnan, Muhammad Noor

**Affiliations:** 1 Department of Biotechnology, Quaid-i-Azam University, Islamabad, 45320 Pakistan; 2 College of Plant Sciences and Technology/National Key Laboratory of Crop Genetic and improvement, Huazhong Agricultural University, Wuhan, Hubei 430070 P. R. China; 3 State Key Laboratory of Agricultural Microbiology and College of Veterinary Medicine, Huazhong Agricultural University, Wuhan, Hubei 430070 P. R. China; 4 School of Biotechnology, Jiangnan University, Wuxi, 214122 P. R. China; 5 Department of Agriculture, University of Swabi, Swabi, Khyber Pakhtunkhwa Pakistan; 6 Zhejiang University Hangzhou, Hangzhou, 310058 People’s Republic of China; 7 Department of Zoology, Kohat University of Science and Technology, Kohat, 26000 Pakistan; 8 Pakistan Academy of Sciences, Islamabad, Pakistan; 9 Institute of Chemical Sciences, Gomal University D.I.Khan, 29050 KPK, Pakistan; 10 Inner Mongolia University, Huhot, 010021 P. R. China; 11 School of Chemistry and Chemical Engineering, Sun Yat- Sen University, Guangzhou, 510275 P. R. China

## Abstract

*Atropa acuminata Royle Ex Lindl* (*Atropa acuminata*) under tremendous threat of extinction in its natural habitat. However, the antimicrobial, antileishmanial and anticancer effects of the plant’s extracts have not been reported yet. In the current study, an attempt has been made to evaluate the pharmacological potential of this plant’s extracts against microbes, Leishmania and cancer. The roots, stems and leaves of *Atropa acuminata* were ground; then, seven different solvents were used alone and in different ratios to prepare crude extracts, which were screened for pharmacological effects. The aqueous, methanolic and ethanolic extracts of all parts carried a broad spectrum of anti-bacterial activities, while no significant activity was observed with combined solvents. Three types of cytotoxicity assays were performed, i.e., haemolytic, brine shrimp and protein kinase assays. The aqueous extract of all the parts showed significant haemolytic activity while n-hexane extracts of roots showed significant activity against brine shrimp. The acetone extracts strongly inhibited protein kinase while the methanolic extracts exhibited significant cytotoxic activity of roots and stem. The anti-leishmanial assays revealed that the methanolic extract of leaves and roots showed significant activity. These findings suggest that this plant could be a potential source of natural product based drugs.

## Introduction

According to the World Health Organization (WHO), approximately 80% of the world’s population use medicinal plants for their primary health care, and herbal remedies have been widely used for the treatment of many fatal human diseases in the past decade^1,2^. *Atropa acuminata Royle Ex Lindl* (*A. acuminata*), an important medicinal plant, belongs to family *Solanaceae*. It is endemic to northern Pakistan, Kashmir and India and is commonly known as Indian Belladonna. In the Himalaya region, it is mostly found in western regions of the subcontinent, starting from Kashmir at the altitude of 1.8–3.6 kilometres (km) to the connecting hills of the Himachal Pradesh up to 2.5 km in altitude. In Northwest Himalaya, it is reported from Kashmir, Muzaffarabad and Chakrata^3–5^.

In traditional medicines, the rhizome and aerial parts of the plant have been used for ages to relieve joint pain, muscle pain, and muscle spasms and to treat arthritis, pancreatitis, peritonitis, scarlet fever, Parkinson’s disease and neuro disorders^6,7^. Recently, the ethanolic extract of the plant has been reported to possess anti-arthritic activity^8^. The plant is the best source of tropane alkaloids such as atropine, hyoscyamine and scopolamine, which possess anticholinergic activity and have diversified therapeutic uses in the fields of ophthalmology, cardiology, and gastroenterology^9,10^. *A. acuminata* showed strong anti-inflammatory activity in carrageen an-induced inflammation mouse models^11^. Traditionally, the leaves and roots are used as relaxants, diuretics, mydriatics, narcotics and sedatives^12^.

The main constituents of *A. acuminata*. are monoterpenes, sesquiterpenes, phenyl propanoids, flavonoids, saponins and quinine^13^. *A. acuminata* also contains tropane alkaloids and highly oxygenated triterpenes^14^. The analysis of hairy root culture of *Atropa* through Direct Analysis in Real Time (DART) spectrometric technique revealed that it contains a high amount of alkaloids. High-performance liquid chromatography (HPLC) analysis of different parts of *Atropa* reveals that each has different alkaloid contents^15^. The leaves contain an average of 0.4% active alkaloids, whilst the root contains round 0.96%. The alkaloid contents also vary according to the age and developmental stage of the plant. At early ages, it contains low amounts of alkaloids, but the contents increase at the flowering stage^16^. According to a report, the ethanolic extract from the leaves of the plant contains approximately 188 micro grams of phenolic compounds per ml extract, which is a huge amount compared to other plants in the family^17^.

Since *A. acuminata* under tremendous threat of extinction in its natural habitat, the current study was designed to accomplish the *in vitro* pharmacological evaluation of various organic extracts of the plant by assessing different types of biological activities. This research will draw attention to the biological conservation of the plant as well as increase our understanding of the active ingredients in *A. acuminata*. However, further research is needed to elucidate the molecular and biochemical mechanisms acting against these microbes, cancer and Leishmania. In addition, this research may also provide a platform for further studies on *A. acuminata* regarding its pharmacological and clinical application in the treatment of leishmaniasis and cancer.

## Results

### Anti-microbial Activity

#### Antibacterial assay

Crude extracts of *A. acuminata* were evaluated against seven bacterial strains. Among them, five were gram negative, and two were gram positive. The minimum inhibitory concentration of each extract is given in Table 1. Among the single solvents, the best activity is shown by the aqueous extract, followed by the methanolic and ethanolic extracts, while no significant activity was seen for polar solvents except chloroform, with higher MICs for each strain. The same pattern was seen for combined solvents with no antagonistic or synergistic effects.

**Table 1:** Anti-bacterial activities of 42 extract from stems, roots and leaves of *A. acuminata*, (—) represents no activity of the respective samples.

Plant Parts	Bacterial Species	Crude Extracts														
		n-Hexane	Chlorophom	Ethyl acetate	Acetone	Ethanol	Methanol	Water	n-Hexane: Ethyl acetate	Ethanol: n- Hexane	Methanol:Chloroform	Methanol: Ethyl acetate	Methanol: Acetone	Acetone: Water	Methanol: Water	Cefotaxime sodium
**Leaves**	*B. Subtillus*	—	26.3	—	—	11.34	11	9.13	—	18.65	12	13.4	34	26.4	9.3	7.9
	*E. coli*	—	21	—	—	13	11	11.23	—	14	38.3	10.7	38	12	10	7.3
	*K. Pneumonie*	—	21.4	—	—	13.45	19	14.56	—	—	14.8	11	18	12	14.2	8.4
	*M. Luteus*	—	11	39	39	30	21	12.87	12.87	—	16.2	16.37	13.45	13.43	11	6.7
	*P. aeruginosa*	—	23	—	—	10	28.5	18.24	—	12.7	15.4	11	37.9	16.25	23	8.83
	*S. Typ*	—	36.5	—	—	13	18	21	—	27	25.2	13.4	15.24	18.35	19	6.58
	*S. aureus*	—	40	37	37	—	12.35	14	—	26.3	—	17	18	12.87	13.24	8.13
**Stems**	*B. Subtillus*	—	23	—	—	12	14.4	14.53	—	13.7	17.6	36	18	23	23	
	*E. coli*	—	21.9	—	—	29	11.56	10	—	27	28.4	10.13	33	18	62	
	*K. Pneumonie*	—	35	—	—	—	17.25	19.4	—	39.3	—	17.2	24	26.53	11	
	*M. Luteus*	—	16	—	—	26	20.11	17.62	17.62	20	21.52	29	18.6	10.84	14.17	
	*P. aeruginosa*	—	21	—	—	17.6	19	9.47	—	17	23.2	26	—	28.78	15.4	
	*S. Typ*	—	—	—	—	17.27	11.32	26.3	—	5	13	15.71	17	17	11	
	*S. aureus*	—	—	—	—	26.12	20	21.5	—	—	26.3	12	21.8	13.9	16	
**Roots**	*B. Subtillus*	—	13.84	—	—	9.18	10.38	10.46	—	18.3	9.34	24.6	23	9.15	12	
	*E. coli*	—	16.2	—	—	11.36	11	9.93	—	17.6	14.17	17	13	14	13	
	*K. Pneumonie*	—	13.3	—	—	11.54	14.51	11	—	14.8	19.7	10.7	9	13	17	
	*M. Luteus*	—	10.3	—	—	9	10.45	10	10	22.8	19.1	16.43	14	22	9.35	
	*P. aeruginosa*	—	14	—	—	13.5	18.27	24.56	—	20.3	9.26	9.73	12	26	11	
	*S. Typ*	—	15.21	—	—	9	13.43	12.4	—	19.2	16.13	13	9	21	13.6	
	*S. aureus*	—	22	—	—	14.86	9.74	11.8	—	18	15.82	15	23	19	11	

The anti-bacterial activity of 42 extracts from roots, stems, and leaves showed a diverse class of results. However, broad-spectrum results were shown by the aqueous methanol, ethanol and chloroform extract^18^. The aqueous extract from stems showed a somewhat lower MIC range than that from leaves and roots. This clearly demonstrates the fact that the roots of these plants contain steroidal saponins that are less hydrophilic, but organic polar solvents such as methanol, ethanol and some phenolic solvents have a greater affinity towards them^19^. Nevertheless, some plants have low levels of steroidal saponins in their leaves, but still, their extract has higher antibacterial activity due to some flavonoids and phenolic compounds^20,21^. In conclusion, these results revealed that each of the three parts of *A. acuminata* contains active compounds that have a broad spectrum of antibacterial activities; however, the principal compound isolation and structural elucidation have yet to be achieved.

#### Anti-Fungal Assay

Anti-fungal activity was determined against four different strains of fungi, i.e., *Aspergillus*, *Fumigatus, Flavous* and *Mucor*. Among the single solvents, the best activity was shown by mostly polar solvents, although in non-polar solvents, no satisfactory anti-fungal activity was observed, except for in chloroform, which exhibited slight activity in combined solvents. The acetone-water extract had the best activity compared to aqueous or acetone extract separately as shown in Table 2, which means that the acetone-water combination enhanced the activity of the extract, although no antagonistic or synergistic effects were seen in the remaining combined solvent systems.

**Table 2:** Anti-fungal activity of 42 extracts from stems, roots and leaves of *A. acuminat*a extracts, zone of inhibition in (mm), ±standard deviation while character (—) is the representative of no activity for that extract against the fungal specie.

Solvents	Fungal Species	Plant Parts											
		Leaves				Stems				Roots			
		Aspergilus-fumigatus	Candida albicans	Aspergillus-flavus	Mucor indicus	Aspergilus-fumigatus	Candida albicans	Aspergillus-flavus	Mucor indicus	Aspergilus-fumigatus	Candida albicans	Aspergillus-flavus	Mucorindicus
n- Hexane		7 ± 1	9 ± 0.7	—	13 ± 2.31	7 ± 3	3 ± 0	7 ± 1.35	11.2 ± 0.33	5 ± 0	9 ± 0.53	—	11 ± 0
Chloroform		8 ± 0.8	9.34 ± 1	7 ± 1.56	20 ± 1.3	9.1 ± 1.6	9.54 ± 1	8 ± 2	12.35 ± 1.6	23.2 ± 4	11 ± 2	12.5 ± 1	14.66 ± 0.38
Ethyl acetate		7 ± 0	7 ± 0.14	5 ± 1	11.3 ± 0.6	3 ± 1	8 ± 0.2	10 ± 1	10 ± 0.8	11 ± 1	8 ± 0	10 ± 0.55	8 ± 1
Acetone		5.5 ± 1	9 ± 1	5 ± 0.71	14.42 ± 2	11 ± 1	9 ± 0.45	10 ± 1.62	10.21 ± 0	13 ± 0	9 ± 0.4	10 ± 2	10.23 ± 1
Ethanol		—	9.6 ± 1	—	14 ± 1.7	13.5 ± 0.4	3 ± 0.28	7 ± 1	8 ± 1.32	9 ± 0	11.5 ± 0.6	3 ± 0.21	11 ± 1.56
Methanol		7 ± 0	10 ± 1.24	9 ± 0.87	7 ± 1	7 ± 0	—	9 ± 0.36	3 ± 0	10 ± 0	3.2 ± 0	8 ± 1	8.06 ± 0.35
Water		—	8 ± 0	—	13 ± 0	1.5 ± 0	—	7 ± 0	5 ± 0.34	—	9.13 ± 1	7 ± 0	10.71 ± 1
n-Hexane: Ethyl acetate		—	8 ± 2.23	—	11 ± 1	4 ± 1	8 ± 1	—	11 ± 2	3 ± 0	3 ± 0	—	8 ± 0
Ethanol: n- Hexane		7 ± 2	15 ± 1	5 ± 0	13.2 ± 1	5 ± 2	—	—	9 ± 1.26	10 ± 0	—	8 ± 1.42	7 ± 0.64
Methanol: Chloroform		3 ± 1	9 ± 1.83	8 ± 1.3	9 ± 2	9 ± 0	7 ± 0.34	5 ± 0.53	12 ± 1	8 ± 1	6 ± 1	7 ± 1.7	8 ± 1.52
Methanol: Ethyl acetate		7 ± 0	12 ± 2	9 ± 0	11 ± 0.43	10 ± 2.53	8 ± 2	6 ± 1.6	10 ± 0.7	5 ± 0	10 ± 0.33	—	7.4 ± 1
Methanol: Acetone		8 ± 1	10 ± 1	5 ± 0.4	13.57 ± 2	5 ± 1.4	8 ± 0.13	10 ± 1.71	—	13 ± 0	10.8 ± 2	10 ± 2.27	8 ± 1.46
Acetone: Water		10 ± 0.78	18 ± 0.73	5 ± 1	14 ± 2	4 ± 1.3	—	5 ± 0	—	—	13 ± 1.76	—	—
Methanol: Water		3 ± 0	7 ± 0	—	14 ± 1.67	9 ± 0	6 ± 1	8 ± 0.31	9.43 ± 2	3 ± 0	14 ± 2.17	—	8 ± 2
Fluconazole		25 ± 13	18 ± 7	16 ± 4	21 ± 39								

### Cytotoxic activity

#### Haemolytic assay

Haemolytic assays were performed for all extracts of each part (root, stem, and leaves). Values above 500 µg/ml were taken as non-significant and represented by a dash, while values lower than 50 µg/ml were considered to indicate strong cytolysis activity^22,23^. The aqueous extracts carried the lowest IC50 values, while the chloroform extract showed the second strongest activity. Acetone, methanol: ethyl acetate and acetone: water extracts also showed the best activity, as evident from Table 3. The ethyl acetate, methanol, ethanol and methanol: chloroform extracts showed non-significant values; however, good synergistic activity was seen in the methanol: ethyl acetate combined solvent system.

**Table 3:** Hemolytic activity of the leaves extracts.

S. No	Extracts	50 µg/ml	100 µg/ml	150 µg/ml	200 µg/ml	IC50 µg/ml
1	n-Hexane	88.37	94.59	101.34	135.64	284
2	Chloroform	93	106.59	126.89	121.27	14*
3	Ethyl acetate	100	105.51	108.96	115.42	**>500**
4	Acetone	78.59	100.55	115	117.42	22*
5	Ethanol	74.02	90.42	92.47	111.70	**>500**
6	Methanol	73.95	90.93	96.27	111.70	>**500**
7	Water	72.53	88.83	109.49	116.57	13*
8	n-Hexane: Ethyl acetate	73.40	94.27	194.68	221.93	28*
9	Ethanol: n- Hexane	81.38	94.15	97.27	196.34	239
10	Methanol: Chloroform	72.34	77.13	87.76	100.42	**>500**
11	Methanol: Ethyl acetate	49.21	85.17	96.64	161.17	32*
12	Methanol: Acetone	78.95	142.72	190.74	345.78	294
13	Acetone: Water	60.11	80.85	122.87	137.23	36*
14	Methanol: Water	47.34	75	76.59	91.49	52
15	Triton-X100	123	158	189	215	5

The non-significant values are shown in bold while the *represents the most significant values.

The ethyl acetate presented the best haemolytic activity among the stem extracts, while methanol and acetone showed the second and third highest significant activity. The remaining 11 extracts fell within the significant range (below 500). In comparison with the leaf extracts, no synergism was seen in combined solvent systems, yet the methanolic extract was seen to shift from non-significant (in leaves) to the second most significant one (in the stem), as shown in Table 4.

**Table 4:** Hemolytic activity of the stem extracts, the *represent the most significant values.

S. No	Extracts	50 µg/ml	100 µg/ml	150 µg/ml	200 µg/ml	IC50 µg/ml
1	n-Hexane	37.23404	78.85106	80.57447	113.8298	64
2	Chloroform	56.42553	90.93617	111.1702	145.7447	39
3	Ethyl acetate	61.17021	75.4805	89.74468	105.1064	14*
4	Acetone	58.10638	82.97872	82.31915	85.6383	49*
5	Ethanol	81.91489	136.7021	136.8511	383.5106	223
6	Methanol	57.7234	70.65957	90.31915	124.6596	33*
7	Water	39.7234	78.14894	82.97872	92.34043	55
8	n-Hexane: Ethyl acetate	76.59574	102.766	112.6596	197.3404	260
9	Ethanol: n-Hexane	37.53191	90.85106	96.2766	105.0213	53
10	Methanol: Chloroform	73.93617	96.80851	111.1702	209.5745	247
11	Methanol: Ethyl acetate	37.19	53.87234	67.19149	78.02128	86
12	Methanol: Acetone	47.087	68.29787	79.7234	88.78723	54
13	Acetone: Water	41	68.87234	72.14894	84.04255	59
14	Methanol: Water	16.59574	60.38298	78.89362	89.7234	83
15	Triton-X100	123	158	189	215	5

Among the root extracts, only three, i.e., ethanol, ethanol: n-hexane and acetone: water, revealed higher IC50 values, while the remaining exhibited good cytotoxic activity, of which the best three were methanol, acetone and n-hexane has been shown in Table 5.

**Table 5:** Hemolytic activities of the root extracts, the *represent the most significant values.

S. No	Extracts	50 µg/ml	100 µg/ml	150 µg/ml	200 µg/ml	IC50 µg/ml
1	n-Hexane	98.93617	195.7447	223.4043	241.4894	43*
2	Chloroform	40.14894	87.87234	98.34043	173.4043	52
3	Ethyl acetate	18.08511	99.46809	115.4255	150.3191	61
4	Acetone	50.10638	85.6383	85.6383	137.234	19*
5	Ethanol	78.12766	112.8085	125.6383	169.1489	400
6	Methanol	88.82979	99.95745	100.0426	109.0638	16*
7	Water	52.59574	113.8936	120.7447	132.9787	49
8	n-Hexane: Ethyl acetate	66.57447	109.4894	114.8936	164.8936	16
9	Ethanol: n- Hexane	77.12766	86.70213	89.89362	122.3404	283
10	Methanol: Chloroform	43.30813	75.48936	91.80851	92.02128	56
11	Methanol: Ethyl acetate	26.97872	80.31915	85.10638	90.95745	57
12	Methanol: Acetone	47.14894	69.97872	72.34043	90.95745	54
13	Acetone: Water	78.7234	95.21277	60.10638	73.40426	322
14	Methanol: Water	21.85106	66.68085	74.02128	81.91489	69
15	Triton-X100	123	158	189	215	5

#### Brine shrimp lethality assay

Brine shrimp lethality assays were performed for those extracts having the lowest IC50 values in haemolytic assays^24^. For this purpose the top three extracts in terms of good IC50 values were taken from each part, i.e., three each from roots stems and leaves. The lethal dose_50_ (LD50) is the lowest concentration of a drug that has the ability to kill 50% of the cells^25^. The root methanolic extracts showed the lowest LD_50_ values while the highest was observed for n-hexane. Similarly, among the stem extracts, the best activity was shown by ethyl acetate followed by aqueous extract, but no significant value was noted for the acetone extract. Among the leaf extracts, the methanolic extract revealed an LD_50_ value of 61 µg/ml and the acetone extract showed a non-significant value, whereas the chloroform extract showed good activity. Hence, it was observed that overall the methanolic extracts of roots and leaves had the same metabolites, which have potent cytotoxic activity (Table 6).

**Table 6:** The brine shrimps lethality assay of 9 extracts of three different parts of *A. acuminata*, the suffix *shows very low activity while the (—) shows that the LD50 is more than 500 µg/ml.

Extracts	5 µg/ml	10 µg/ml	50 µg/ml	100 µg/ml	LD50 µg/ml
**Roots**					
Acetone	0	0	1	7	136
Methanol	0	0	3	6	66
n-Hexane	0	1	1	3	478*
**Stems**					
Acetone	0	0	0	2	—
Ethyl acetate	0	0	3	5	100
Water	0	1	3	4	178
**Leaves**					
Acetone	0	0	0	1	—
Chloroform	0	0	1	5	108
Methanol	0	2	3	7	61

#### Protein Kinase inhibition Assay (PK)

A protein kinase inhibition assay was also performed for those extracts that had the lowest IC50 values in the haemolytic assay^26^. In plate reading three different types of readings were observed, cloudy zones, clear zones or no zones. Clear zones of inhibition were observed for methanol extracts of roots and leaves, cloudy zones for acetone extracts of stem and roots and no zones for aqueous extract of stems. The 13 ± 2 mm and 9 ± 1 (Table 7) zones of inhibition for the methanol extracts of leaves and roots show that they have strong cytotoxic activity.

**Table 7:** Protein kinase inhibition assay: (—) means not observed for particular result.

Extract	Clear zone	Cloudy zone	No zone
**Roots**			
Acetone	—	Yes	—
Methanol	9 ± 1 mm	—	—
n-Hexane	—	Very small	—
**Stems**			
Acetone	—	Yes	—
Ethyl acetate	4 ± 1 mm	—	—
Water	—	—	—
**Leaves**			
Acetone	—	Very small	—
Chloroform	4 mm	—	—
Methanol	13 ± 2 mm	—	—

#### Anti-leishmanial Assay

An anti-leishmanial assay revealed that the methanolic extract from either leaves or stem was active against both promastigote and amastigote parasites. However, a shift was observed in the IC50 values for both parasites. This shift was calculated in terms of the ratio of the IC50 for promastigotes to the IC50 for amastigotes^27^. The shift for the methanolic extract from roots was 0.94, while for the root extract, it was 0.6; this means the methanolic extract from the roots is comparatively more active against promastigote than against amastigote and vice versa. The same pattern was seen for acetone extract from roots, chloroform extract from leaves, and ethyl acetate extract from stems; where the SI value for acetone extract from leaves exceeded 1, it means the pattern was the opposite, i.e., more active against amastigote then promastigote. The aqueous and acetone extracts from stems showed no significant activity (Table 8).

**Table 8:** The anti-leishmanial activities of different extracts from *Atropa acuminata*, the symbol ∞^A^ means no significant activities and B represent the ratio of IC 50 for anti-promastigote to the IC 50 for anti-amastigote.

Extract	Part of plant used	anti-Promastigotes Activity IC50 µg/ml	anti-Amastigote Activity IC50 µg/ml	SI^B^
Acetone	Roots	136	147	0.92
Methanol		88	93	0.94
n-Hexane		493	456	0.1
Acetone	Stems	318	>500	∞^A^
Ethyl acetate		252	439	0.57
Water		368	>500	∞^A^
Acetone	Leaves	198	176	1.1
Chloroform		126	133	0.94
Methanol		53	77	0.6
Amphotericin B		46	57	0.80

## Discussion

### Antimicrobial Assays

Both the antibacterial assay, the anti-fungal assay showed an opposite pattern of activities among the extracts. For example, the aqueous fraction from all the parts showed a broad spectrum of anti-bacterial activities MIC ranges from 9.1 µg/ml against *B. subtillus* to 26.3 µg/ml against *S. type* (Table 1). When tested against fungi the same fraction showed 0 mm (leaves), 1.5 mm (stem) and 0 mm (roots) zones of inhibition against *Aspergilus fumigatus* while 13 ± 0.4 mm, 5 ± 0.34 mm and 10.71 ± 1 mm zones of inhibitions were noted against *Mucor indicus* for leaves, stems and roots respectively (Table 2). Conversely, no significant difference was found in antibacterial effects between extracts from roots, stems and leaves except the chloroform extract from the stems that showed no significant effects against *S. type* and *S. aureus*. This means that all of the three parts carried almost the same properties for the extracts that showed the best anti-bacterial activity^28^. However, in the anti-fungal assay, only the chloroform, acetone and ethyl acetate fractions showed a broad spectrum of anti-fungal activity irrespective of the part used^29,30^. These activities are mainly due to tannins, some non-polar terpenoids or most likely some phenols, flavonoids and hydrophobic saponins. However, their exact identification is still in infancy^31,32^.

### Cytotoxic assays

Cytotoxic assays of the different extracts from *A. acuminata* revealed the same pattern of activities with different IC50 values except for a few extracts, the IC50 values of which were not in the acceptable range (more than 500 µg/ml). Similarly, some extracts showed the lowest minimum inhibitory concentrations in the haemolytic assay, which led us to subject them to further evaluation.

The IC50 calculation method is more acceptable and accurate, so we used the term IC50 to describe our results^33^. The comparative analysis of all the extracts from different parts shows that the aqueous extract from leaves showed the lowest IC50 value followed by chloroform extract from the same part, ethyl acetate from the stem and acetone from the roots. This describes the fact that each part contains a different active agent from a class of compounds or different compounds from the same class with different affinities towards the same solvent. However, this may be confirmed by high-performance liquid chromatography (HPLC) or gas chromatography (GC) analysis.

The brine shrimp lethality assays showed a quite simple pattern of results. The methanol extract from roots and leaves showed the lowest LD50, while the results shown by acetone extracts from all the three parts were not satisfactory. The remaining extracts presented different values in each part. Although the selection of all these extracts for brine shrimp assays was made on the basis of their potential activity in the haemolytic assay (see results section), the outcome in the brine shrimp assays appeared quite different. This clearly deviates from the hypothesis that all haemolytic compounds will also destroy the cells of brine shrimp and supports the studies in which the methanol extracts of many plants contain strong killers of brine shrimp^34,35^.

### Protein Kinase Assay

The PK assay showed the same pattern of results for cell killing as the brine shrimp assay except for the acetone extract, which showed inhibition of the enzymes^36^. The methanol extracts from both roots and leaves showed that these extracts of *A. acuminata* have a broad spectrum of cytotoxic activities against a variety of cells and can be used in future not only for cancer therapy but also to kill many parasitic insects^37^.

In conclusion, all three parts of *A. acuminata* showed a diverse range of cytotoxic activities; however, a good correlation was found among the methanolic extracts in all the assays, which demonstrates that the methanolic extract of this contains a potent cytotoxic active agent to be reported upon further analysis.

### Anti-leishmanial assay

As plants become a valuable source of new medicinal agents in the absence of vaccines, we calculated the infection kinetics of different extracts of *A. acuminata*. Remarkable results were obtained for both promastigote and amastigote parasites. Our results showed that the methanol fraction from leaves have IC50 of 53 µg/ml and 77 µg/ml for promastigote and amastigote respectively which is more near to 46 µg/ml and 57 µg/ml for the currently clinically used (Ampoteicine B). When the ratio of promastigote to amastigote IC50 values was calculated we found different efficiencies of different parts of *A. acuminata* extracts in different solvents ranges from 0.57 to 1.1. As a future perspective, the constituents of different parts of the plant can be characterized to identify active compounds responsible for the positive activity so that it can be used to treat infections caused by parasites.

## Conclusion

The current study investigated *A. acuminata* is a pharmacologically active plant. Many extracts from leaves, stems and roots have broad-spectrum biological activities, but leaves have more potential than other parts. These findings suggest that the plant could be a potential source for infectious diseases caused by microbes and parasites. Cytotoxic results show that this plant extract may show good inhibition of cancerous cells and tumours, so this research provides a baseline for further studies, and further chemical and clinical analyses are needed to investigate the correct structure and mechanism of action of the active agent. Moreover, protection measures concerned with the conservation and cultivation of this medicinally important plant are also needed.

## Materials and Methods

### Collection of plants and solvent extraction

*A. acuminata* plant parts (leaves, stems and roots) were collected from the hilltops of Toormang Valley, Dir (Upper), Khyber Pakhtunkhwa, Pakistan. A herbarium specimen with a voucher number (KAA-22) was submitted to the herbarium library of Molecular Systematics and Applied Ethnobotany Lab, Department of Biotechnology, Quaid-i-Azam University, Islamabad, Pakistan, and identified by a taxonomist. The plant parts were dried and ground into fine powder for preparation of extracts. The powders of each part were taken in labeled flasks in the equal concentration of one milligram of powder per three millilitres of solvent (1 mg/3 ml) (25 mg of each part powder in 75 ml of each solvent respectively). Seven different solvents and their combinations were used (Table 9). After adding the powder and solvents, the flasks were tightly closed and sonicated at 60 kHz for 10 minutes to rupture the cells (so that maximum quantity of metabolites will ooze out) and kept for 2–3 days to extract maximum metabolites, that were finally filtered through cellulose filter paper. The extracts were dried in large petri plates and stored in sterilized bottles. A stock solution of each extract was prepared by dissolving 4 mg crude extract in 1 ml absolute DMSO (Dimethyl Sulfoxide) for further activities.

**Table 9:** Names of the solvents used alone and as binary mixtures, and the ratios.

No	Solvents	Solvents Ratio	Powder Weight in grams	Solvent and plant powder Ratio volume/weight
1	n-hexane	—	30	3:10
2	Chloroform	—	30	3:10
3	Ethyl acetate	—	30	3:10
4	Acetone	—	30	3:10
5	Ethanol	—	30	3:10
6	Methanol	—	30	3:10
7	Water	—	30	3:10
8	n-hxane: Ethyl acetate	1:1	30	3:10
9	ethanol: n- hexane	1:1	30	3:10
10	methanol: chloroform	1:1	30	3:10
11	methanol: Ethyl acetate	1:1	30	3:10
12	methanol: acetone	1:1	30	3:10
13	aceton: water	1:1	30	3:10
14	methanol: water	1:1	30	3:10

### Evaluation of Anti-microbial Activity

#### Antibacterial assay

Anti-bacterial tests were performed for each extract against seven bacterial strains i.e. *Bacillus subtillus* (ATCC 6051-U)*, Escherichia coli* (ATCC 33559), *klebsiella pneumoniae* (ATCC BAA-1705), *Micrococus luteus* (ATCC 12698), *Pseudomonas aeruginosa* (ATCC BAA-2110), *Staphylococcus aureus* (ATCC 35844), and *Salmonella typhi* (ATTC 39926) (Table 1). The broth dilution method was used to assess antibacterial activity. In brief, the strains were cultured on LB (Luria Bertani) broth (is the most widely used medium for bacterial growth, in liquid form, it is known as LB broth) medium for 12 hours followed by cold shock at 5–3 °C. Then, each culture was diluted, and to test the extracts against bacterial culture, 7.5 µl of crude extract was taken from 4 mg/ml DMSO stock extract in 96-well plates and serially diluted four times with DMSO to obtain 4 different concentrations. After making the dilutions, 195 µl of inoculum was added to each well^38^. Cefotaxime sodium was taken as a positive control in the same concentration as the extract, while 5 µl of DMSO was taken as a negative control. Readings were taken two times on a micro plate reader (ELx 800 BioTek) at a wavelength of 630 nm. The first or R_1_ reading was taken just after a half hour of incubation, while the second reading or R_2_ was taken 22 hours after R_1_. The percent growth inhibition was calculated using the formula^39^:

1 $${\rm{Percent}}\,{\rm{Inhibition}}=({{\rm{R}}}_{{\rm{2}}}-{{\rm{R}}}_{{\rm{1}}})/({\rm{C1}}-{\rm{C2}})\times 100$$

Where

R2 = Sample reading after 22 hrs.

R1 = Sample reading at 0 hrs.

C2 = Control reading after 22 hrs.

C1 = Control reading at 0 hrs.

#### Antifungal assay (MIC, MFC)

Old cultures of all selected fungal strains i.e. *Aspergilus fumigatus* (ATCC 1022), *Candida albicans* (ATCC MP-8), *Mucor indicus* (ATCC 90364), *Aspergillus flavus* (ATCC 16883) were re-cultured on tryptic soy broth (TSB) medium for 24 hours to obtain active and fresh cultures. After 24 hours, the refreshed cultures were spread on Sabouraud dextrose agar (SDA) medium using a sterilized bent glass rod. The disc diffusion method was used to check the antifungal effect of the crude extracts at the concentration of 20 mg/ml, and discs were placed on their respective labelled plate for 50 to 70 hours at 25 to 30 °C. Clotrimazole (4 mg/ml) stock was used as a positive control.

### Anti-leishmanial Activity

#### Anti-Promastigote Activity

For this assay, a culture of *Leishmania major* (KMU 25) was sub cultured in RPMI 1640 medium (Bio-Rad) with 10% foetal bovine serum (FBS) added. Then, the culture was incubated for 6 days, subjected to light microscopy and counted using a haemocytometer at 2.5 × 10^6^ cells/ml density. The anti-promastigote assay was performed using an MTT (3-(4, 5-dimethylthiazol-2-yl)-2,5-diphenyltetrazolium bromide)-based micro-assay as a detector of cell sustainability^40^. Ten microlitres of each crude extract from 20 μg/ml stocks and 100 μL from a culture containing 2.5 × 10^5^ cells per ml were added to 96-well cell culture plates. The plates were then incubated at 24 °C for three days. DMSO was used as negative control, and amphotericin was taken as positive control. Then, 10 μl of MTT reagent was added to each well, and the plates were re-incubated under the same conditions for at least 3 hours. Then, 100 μl of a solution containing 50% isopropanol and 10% SDS (sodium dodecyl sulphate) was added in the same pattern and the incubation step repeated for a half hour. Then, readings were taken at 540 nm using (ELX 800, BIOTEK) a micro-titre plate reader. The percent viability of cells was calculated using the formula:

2 $${\rm{Percent}}\,{\rm{Viable}}\,{\rm{cells}}=[({{\rm{A}}}_{{\rm{e}}}-{{\rm{A}}}_{{\rm{b}}})/({{\rm{A}}}_{{\rm{c}}}-{{\rm{A}}}_{{\rm{b}}})]100$$

Where A_e_ is the absorbance of the plant extract, A_c_ is the absorbance of the control and A_b_ is the absorbance of the blank. Efficient fractions were selected on the basis of % mortality for further processing. The IC50 (% inhibitory concentration) for each extract was calculated on the basis of threefold serial dilution^41^.

#### Anti-amastigote Activity

The Amastigote culture was donated by Dr. Yaseen Yousafzai, laboratory of Nano-technology Quaid-i-Azam University Islamabad Pakistan, preserved in M119 medium (pH 5). One millilitre of the leishmanial culture was centrifuged for 10 minutes at 3000 rpm, and the pellet was removed and washed with PBS at least three times. The pellet was then transferred into 20 ml of medium and incubated at 33 °C. 15 μl of each crude extract from the 4 mg/ml stock was added to the first 14 wells in a row order from left to right and then serially diluted in the consecutive rows to obtain 4 different concentrations of each extract, i.e., 65 µg/ml, 130 µg/ml, 261 µg/ml and 546 µg/ml. Then, 100 μL of the culture containing 2.5 × 10^6^ cells/ml promastigotes was added to each well. The plates were kept in an incubator for 70–74 hours at room temperature. DMSO was used as a negative control and amphotericin as used as a positive control. After incubation, 10 μL of MTT reagent was added to each well and re-incubated for 3 hours under the same conditions. The rest of the procedure was the same as for promastigotes^41^.

#### Evaluation of *in vitro* protein kinase inhibition, cytotoxic and toxic effects

Three assays were performed to assess the protein kinase inhibition, cytotoxic and toxic effects of *A. acuminata* respectively: protein kinase enzyme inhibition assay, haemolytic assay, and brine shrimp assay.

#### Protein kinase enzyme inhibition assay

To evaluate the protein kinase inhibition of the crude extract the method of Hua *et al*., 2005, was used with some modifications^42^. *Streptomyces coelicolor* was used as a test organism, the protein kinase of which has several biochemical and physiological similarities with that of eukaryotes^43^. Two types of media, i.e., tryptic soy broth (TSB) medium and ISP4 (inorganic salt starch agar) medium, were used. A *Streptomyces coelicolor* culture was prepared in TSB medium and then paper discs were dipped in the crude extract at 20 mg/ml. Finally, the plates were incubated at 30 °C for 50 to 70 hours and then readings were taken with the help of a magnifying glass.

#### Haemolytic assay

For haemolytic activity RBCs were isolated from a human. One millilitre of blood was taken and transferred to an EDTA tube followed by centrifugation for 5 minutes at 14000–15000 rpm. The supernatant was then decanted and 800 µl from the remaining pellet (blood) was transferred to 40 ml of phosphate-buffered saline (PBS) and centrifuged for 10 minutes at 2000 rpm. Then, the supernatant was removed and the procedure was repeated three times. Finally, 50 µl was taken from four different stock concentrations i.e., 60 mg/ml, 20 mg/ml, 6 mg/ml, 4 mg/ml and 0.6 mg/ml, of each crude extract. The finally washed RBC pellet was dissolved in PBS, and 150 µl of this suspension was added to the crude extract and incubated for 60 minutes at 37 °C. The suspension was then centrifuged at 2500 rpm for 10 minutes; 100 µl of supernatant from each tube was transferred to a 96-well plate, and cell lysis was checked at 540 nm wavelength using a micro plate reader (ELx 800 BioTek). Triton X-100 (0.5%) was used as positive control, and PBS and DMSO were used as blank controls. The percentage of RBC lysis was calculated using the following formula:

3 $${\rm{Haemolysis}}( \% )=[\begin{array}{c}({\rm{O}}{\rm{.D}}{\rm{.}}\,{\rm{of}}\,{\rm{crude}}\,{\rm{extract}}-{\rm{O}}{\rm{.D}}{\rm{.}}\,{\rm{of}}\,{\rm{simple}}\,{\rm{PBS}})\\ /({\rm{O}}{\rm{.D}}{\rm{.}}\,{\rm{in}}\,{\rm{0}}\mathrm{.5} \% \,{\rm{Triton}}\,{\rm{X}}-{\rm{100}}-{\rm{O}}{\rm{.D}}{\rm{.in}}\,{\rm{PBS}})\end{array}]\times 100$$

#### Toxicity assay against brine shrimp

Toxicity assay of *A. acuminata* against brine shrimp was performed by a method previously described^44^. Seawater was prepared by dissolving 17 g of sea salt in 500 ml of distilled water and filter sterilizing. A compartmentalized rectangular dish made of Teflon was filled with seawater; eggs (Ocean Star Inc., USA) of shrimp (*Artemia salina*) were poured in its large compartment, and the compartment was covered with aluminium foil, while the small compartment was kept bear with a lamp shining on it. These settings were done in an incubator, which was closed and left for 24 hours at 24 °C to 25 °C. After 24 hours, due to the light movement of newly hatched larvae or nauplii was observed from the large compartment to the small compartment, from where they were collected carefully through a dropper into a beaker containing seawater and a small amount of yeast. Different quantities from different stock solutions of each crude extract were poured into a 96-well plate, and 100 ml seawater was added to obtain different concentrations of each extract. Finally, 10–15 nauplii were shifted to each well in the micro plate and observed under a 3 × magnifying glass. The final volume of the mixture was kept at 200 µl in each well by adding further seawater, and it was incubated at 24 °C for 24 hours. The larvae were again picked and counted under a 3 × magnifying glass to check the number of live and dead shrimp in each well. DMSO was taken as a blank control, and the lethal dose (LD50) of the crude extracts for brine shrimp was calculated by applying the formula:

4 $${\rm{D}}={\rm{L2}}-{\rm{L1}}$$

Where

D is the number of dead cells

L1 is the number of live cells before treatment

L2 is the number of live cells after treatment.
