# Author Correction:* In vitro* biological screening of a critically endangered medicinal plant, *Atropa acuminata Royle Ex Lindl* of north western Himalaya

**DOI:** 10.1038/s41598-020-70070-6

**Published:** 2020-09-23

**Authors:** Khaista Rahman, Shahid Ullah Khan, Shah Fahad, Zabta Khan Shinwari, Dilfaraz Khan, Sajid Kamal, Ikram Ullah, Syed Ishtiaq Anjum, Shad Man, Abdul Jamil Khan, Wasim Ullah Khan, Muhammad Hafeez Ullah Khan, Mehmood Jan, Muhammad Adnan, Muhammad Noor

**Affiliations:** 1 State Key Laboratory of Agricultural Microbiology and College of Veterinary Medicine, Huazhong Agricultural University, Wuhan, Hubei 430070 P. R. China; 2 College of Plant Sciences and Technology/National Key Laboratory of Crop Genetic and improvement, Huazhong Agricultural University, Wuhan, Hubei 430070 P. R. China; 3 Department of Biotechnology, Quaid-i-Azam University, Islamabad, 45320 Pakistan; 4 School of Biotechnology, Jiangnan University, Wuxi, 214122 P. R. China; 5 Department of Agriculture, University of Swabi, Swabi, Khyber Pakhtunkhwa Pakistan; 6 Zhejiang University Hangzhou, Hangzhou, 310058 People’s Republic of China; 7 Department of Zoology, Kohat University of Science and Technology, Kohat, 26000 Pakistan; 8 Pakistan Academy of Sciences, Islamabad, Pakistan; 9 Institute of Chemical Sciences, Gomal University, D.I.Khan, 29050 KPK Pakistan; 10 Inner Mongolia University, Huhot, 010021 People’s Republic of China; 11 School of Chemistry and Chemical Engineering, Sun Yat- Sen University, Guangzhou, 510275 People’s Republic of China

Correction to: *Scientific Reports* 10.1038/s41598-018-29231-x, published online 23 July 2018

In the original version of this Article, Affiliations 1 and 3 were not listed in the correct order. The correct affiliations are listed below:

Affiliation 1:

State Key Laboratory of Agricultural Microbiology and College of Veterinary Medicine, Huazhong Agricultural University, Wuhan, Hubei, 430070, P. R. China

Affiliation 3:

Department of Biotechnology, Quaid-i-Azam University, Islamabad, 45320, Pakistan

This error has now been corrected in the PDF and HTML versions of the Article.

